# Nomogram built based on machine learning to predict recurrence in early-stage hepatocellular carcinoma patients treated with ablation

**DOI:** 10.3389/fonc.2024.1395329

**Published:** 2024-05-10

**Authors:** Honghai Zhang, Shugui Sheng, Wenying Qiao, Yu Sun, Ronghua Jin

**Affiliations:** ^1^ Interventional Therapy Center for Oncology, Beijing You’an Hospital, Capital Medical University, Beijing, China; ^2^ Beijing Key Laboratory of Emerging Infectious Diseases, Institute of Infectious Diseases, Beijing Ditan Hospital, Capital Medical University, Beijing, China; ^3^ Changping Laboratory, Beijing, China

**Keywords:** hepatocellular carcinoma, ablation, Lasso-Cox regression, nomogram, recurrence

## Abstract

**Introduction:**

To analyze the risk factors affecting recurrence in early-stage hepatocellular carcinoma (HCC) patients treated with ablation and then establish a nomogram to provide a clear and accessible representation of the patients’ recurrence risk.

**Methods:**

Collect demographic and clinical data of 898 early-stage HCC patients who underwent ablation treatment at Beijing You’an Hospital, affiliated with Capital Medical University from January 2014 to December 2022. Patients admitted from 2014 to 2018 were included in the training cohort, while 2019 to 2022 were in the validation cohort. Lasso and Cox regression was used to screen independent risk factors for HCC patients recurrence, and a nomogram was then constructed based on the screened factors.

**Results:**

Age, gender, Barcelona Clinic Liver Cancer (BCLC) stage, tumor size, globulin (Glob) and γ-glutamyl transpeptidase (γ-GT) were finally incorporated in the nomogram for predicting the recurrence-free survival (RFS) of patients. We further confirmed that the nomogram has optimal discrimination, consistency and clinical utility by the C-index, Receiver Operating Characteristic Curve (ROC), calibration curve and Decision Curve Analysis (DCA). Moreover, we divided the patients into different risk groups and found that the nomogram can effectively identify the high recurrence risk patients by the Kaplan-Meier curves.

**Conclusion:**

This study developed a nomogram using Lasso-Cox regression to predict RFS in early-stage HCC patients following ablation, aiding clinicians in identifying high-risk groups for personalized follow-up treatments.

## Introduction

Primary liver cancer is one of the most common malignant tumors of the digestive system. According to statistics in 2020, the number of newly diagnosed liver cancer cases worldwide reached 905,677, and the number of deaths associated with this malignant disease reached 830,180 (1). Among them, new cases reported in Asia accounted for approximately 72.0% of the total, with China accounting for more than 50% (2, 3). Hepatocellular carcinoma (HCC) is the main subtype of primary liver cancer, comprising around 75% -85% of cases (4). The treatment methods for HCC are diverse and usually customized according to the health status of the patient and the staging of the cancer. Common treatment methods include surgical resection, chemoembolization, anti-angiogenesis therapy, immunotherapy and so on (5–7). Ablation, as an interventional therapy, has been widely used in the treatment of early-stage HCC in recent years. Its emergence provides a new treatment option for HCC patients who are not suitable for surgery or are waiting for liver transplantation. However, although ablation has achieved remarkable results in tumor control, postoperative recurrence remains one of the great challenges in the clinical treatment of HCC (8). Therefore, early detection and prediction of the recurrence risk of HCC patients after ablation treatment are crucial to developing individualized treatment plans and improving patient prognosis.

Currently, the field of HCC treatment and prognosis has attracted extensive academic and clinical interest. However, most studies focused on surgical resection and transarterial chemoembolization (TACE) (9–14), while in-depth studies on ablation therapy are relatively understudied. Although some studies have begun to explore the prognosis of HCC patients after ablation therapy, the clinical practicality and reliability of the results of these studies have been restricted to a certain extent due to the limitations of small sample sizes. Therefore, one of the uniqueness of this study is that we will conduct an in-depth study of the recurrence in patients with early-stage HCC after ablation therapy based on a large-scale patient data set.

Another highlight of this study is the combination of Lasso regression and Cox regression analysis. Cancer research has been a focus of the medical field globally, and to better understand and predict the occurrence of cancer as well as to understand the prognosis of patients, many research teams have established cancer risk scoring systems that combine demographic and clinical data (15–19). However, most of the parameter screening within these still relies on univariate and multivariate analyses, and these traditional methods, when dealing with high-dimensional data, especially when faced with the problem of multicollinearity problems among multiple variables, clearly have their inherent limitations. Lasso regression provides a new perspective on the analysis of high-dimensional data by introducing L1 penalty term, which allows for efficient variable selection and make the model more robust and easier to interpret (20). In this study, we first used Lasso regression to screen for a large number of variables associated with early-stage HCC, and then employed Cox regression to further refine and explain the impact of these key variables on the survival of early-stage HCC patients.

This study aimed to investigate and analyze the factors affecting recurrence after ablation treatment in patients with early-stage HCC, and to construct a recurrence-free survival (RFS) nomogram based on the Lasso-Cox regression model. The nomogram can help clinicians develop more appropriate follow-up strategies and treatment recommendations based on the HCC patient’s specific risk score.

## Materials and methods

### Patients enrolled

This study retrospectively analyzed the data of 898 patients with early-stage HCC who underwent ablation treatment at Beijing You’an Hospital, affiliated with Capital Medical University from January 2014 to December 2022. These patients were diagnosed with early-stage HCC using the diagnostic tools suggested by the American Association for the Study of Liver Diseases (AASLD) (21), including pathological or radiological criteria. The definition of early-stage HCC encompasses either a solitary tumor measuring less than 5cm in diameter or up to three tumors, each having a diameter of less than 3cm (22). All of the patients who were recruited in the study had received ablation therapy, and all experienced a complete response. Complete response was defined as the complete necrosis of lesions confirmed by contrast-enhanced ultrasound, enhanced CT, and/or enhanced MRI, with no areas of enhancement detected within the liver. Patients who were hospitalized between January 2014 and December 2018 were placed in the training cohort (n=565) and those who were admitted between January 2019 and December 2022 were placed in the validation cohort (n = 333).

The inclusion criteria were (i) early-stage HCC patient underwent ablation therapy as the initial treatment method and experienced a complete response; (ii) complete clinical data; (iii) Child-Pugh class A or B; (iv) no extrahepatic metastasis or vascular invasion; (v) no organ dysfunction. The exclusion criteria were (i) secondary liver cancer; (ii) received other treatment before ablation; (iii) loss of follow-up; (iv) severe malnutrition or taking warfarin and other factors that may affect the levels of prothrombin and other indicators to be studied.

The ethics committee granted an exemption for informed consent due to the research being classified as low-risk. The research has received ethical approval from the Ethics Committee of Beijing You’an Hospital, affiliated with Capital Medical University.

### Patients data

The basic characteristic data of patients were collected before ablation treatment. The data mainly includes four types. (i) demographic data: age, gender; (ii) Liver function data: Child-Pugh class, Barcelona Clinic Liver Cancer (BCLC) stage; (iii) imaging examination data: tumor size and number; (iv) laboratory data: lymphocyte (Lym), white blood cell (WBC), platelet (PLT), alanine aminotransferase (ALT), aspartate aminotransferase (AST), total bilirubin (TBIL), direct bilirubin (DBIL), albumin (Alb), Globulin (Glob), γ-glutamyl transpeptidase (γ-GT), prealbumin (Palb), prothrombin time (PT), thrombin time (TT), and fibrinogen (Fib).

### Treatment received

#### Preparations before ablation

Patients should complete relevant examinations before ablation: blood routine, coagulation function, tumor markers, liver and kidney function, infectious disease indicators, electrocardiogram, Computed Tomography (CT) or Magnetic Resonance Imaging (MRI), and B-ultrasound; Based on imaging data, determine the size, location, blood supply, number and so on of the lesions, and design a treatment plan, including needle insertion route, depth, frequency of ablation and so on; Fasting for 12 hours and water deprivation for 4 hours before ablation; Sign the informed consent form for ablation treatment.

#### Ablation procedure

Percutaneous radiofrequency ablation (RFA) treatment was performed by physicians with at least 5 years of experience. In accordance with the contrast-enhanced ultrasound, the position, size, and number of lesions were determined, and the optimal puncture point, puncture path, needle insertion angle, and direction were selected to avoid vital organs, preventing the occurrence of serious adverse reactions. Under ultrasound guidance, the RFA needle was inserted into the center of the tumor. After setting the voltage and time parameters, the ablation procedure was carried out. The radiofrequency range needed to be expanded to 0.5–1cm around the tumor to ensure complete ablation. For patients with a tumor diameter of ≤3.0cm, a single-needle multipoint approach can be employed. However, for those with a tumor diameter >3.0cm, a multi-needle multipoint radiofrequency treatment often yields better therapeutic outcomes. During procedure, changes in the patient’s blood pressure and heart rate were closely monitored. If there was a decrease in blood pressure or an increase in heart rate, the procedure was paused. If the situation stabilized and abdominal bleeding was not observed on ultrasound, the ablation was continued; if necessary, intravenous hemostatic, sedative, and analgesic medications were administered.

### Follow-up

The main endpoint of this study is RFS, which was defined as the time span from treatment to the first recurrence or last follow-up. Within one month after ablation treatment, all patients are required to have a follow-up and re-examination at the outpatient clinic of our hospital. Patients were evaluated for liver function tests, and imaging tests including ultrasound, CT, and MRI. Patients were followed up every 3 months from 2 months to 1 year after ablation treatment, and every six months thereafter. During the follow-up process, if contrast-enhanced CT showed the presence of active lesions within the liver, it was defined as tumor recurrence.

### Statistical analysis

Statistical analysis was performed using R software (version 4.1.3). Continuous variables are expressed as mean ± standard deviation, and categorical variables are expressed as frequency and percentage. The Student’s t-test was used to compare differences between groups in continuous variables, and the Chi-square test was used to compare categorical variables. Lasso is an efficient method for regression on high-dimensional data, and it is used in this study for identifying potential risk variables. The variables that exhibited statistical significance (p < 0.05) were then included in a multifactor Cox regression analysis to further confirm the risk factors associated with HCC recurrence. A nomogram was created in response to the findings drawn from the Lasso-Cox regression. The C-index and the ROC curve were used to evaluate the discrimination of the nomogram, while the calibration curve was utilized in order to ascertain its consistency. DCA was then used to assess the net benefits of the nomogram, with the aim of demonstrating its clinical utility. Patients were categorized into low-, intermediate-, and high-risk groups based on their nomogram scores, and Kaplan Meier plots were used to forecast their recurrence rate.

## Results

### Patients characteristics

This study enrolled a total of 898 early-stage HCC patients who received ablation treatment at Beijing You’an Hospital Affiliated with Capital Medical University from January 2014 to December 2022, of which 565 were included in the training cohort and 333 were included in the validation cohort. The last follow-up time for this study is July 1, 2023 and the median follow-up time was 32.5 months. The baseline characteristic data from the training and validation cohorts were statistically analyzed and the results showed no significant differences between the two groups (P>0.01), suggesting that they could be used for subsequent studies (**Table 1**).

**Table 1:** Characteristic data of the training cohort and validation cohort.

Characteristic	Primary cohort(N=565)	Validation cohort(N=333)	P value
Age	56.48 ± 8.88	56.68 ± 9.23	0.744
Gender (male/female)	457(80.9%)/108(19.1%)	273(82.0%)/60(18.0%)	0.684
Child-Pugh class(A/B)	419(74.2%)/146(25.8%)	263(79.0%)/70(21.0%)	0.103
Cirrhosis (Yes/No)	94(16.6%)/471(83.4%)	46(13.8%)/287(86.2%)	0.260
BCLC stage(0/A)	182(32.2%)/383(67.8%)	120(36.0%)/213(64.0%)	0.241
Tumor number (Single/multiple)	325(57.5%)/240(42.5%)	185(55.6%)/148(44.4%)	0.566
Tumor size (<3cm/≥3cm)	358(63.4%)/207(36.6%)	199(59.8%)/134(40.2%)	0.283
Lym	1.83 ± 0.68	1.85 ± 0.96	0.827
WBC	4.47 ± 2.73	4.24 ± 2.15	0.438
PLT	124.66 ± 40.62	127.32 ± 52.72	0.294
ALT	28.15 ± 23.13	29.09 ± 13.89	0.622
AST	28.23 ± 12.94	30.88 ± 13.89	0.126
TBIL	16.60 ± 10.51	16.34 ± 6.38	0.253
DBIL	23.23 ± 11.12	21.83 ± 4.14	0.562
Alb	36.56 ± 9.36	37.51 ± 4.55	0.833
Glob	28.82 ± 8.67	28.47 ± 4.93	0.491
γ-GT	62.08 ± 26.96	66.96 ± 32.42	0.171
Palb	156.07 ± 63.5	164.54 ± 59.57	0.189
PT	14.55 ± 6.25	14.88 ± 1.43	0.928
TT	16.98 ± 5.86	14.99 ± 1.88	0.217
Fib	2.57 ± 0.12	2.69 ± 0.99	0.129

BCLC, Barcelona Clinic Liver Cancer; Lym, lymphocytes; WBC, white blood cell; PLT, platelet; ALT, alanine aminotransferase; AST, aspartate aminotransferase; TBIL: total bilirubin; DBIL, direct bilirubin; Alb, albumin; Glob, Globulin; γ-GT, γ-glutamyl transpeptidase; Palb, prealbumin; PT, prothrombin time; TT, thrombin time; Fib, fibrinogen.

The average age of the 898 patients included in the study was over 50 years old, and the majority of patients were male, accounting for 81.3%. The training cohort consisted of patients with an average age of 56 years old, with 80.9% of them being male. Similarly, the validation cohort included patients with an average age of 56 years old and 82% were male. 682 patients were Child-Pugh class A, accounting for 75.9% of the total, indicating that most patients had good liver function. 302 patients were at BCLC stage 0 and 596 patients were at BCLC stage A, accounting for 33.6% and 66.4% respectively.

### Lasso-Cox regression analysis

**Figure 1:**
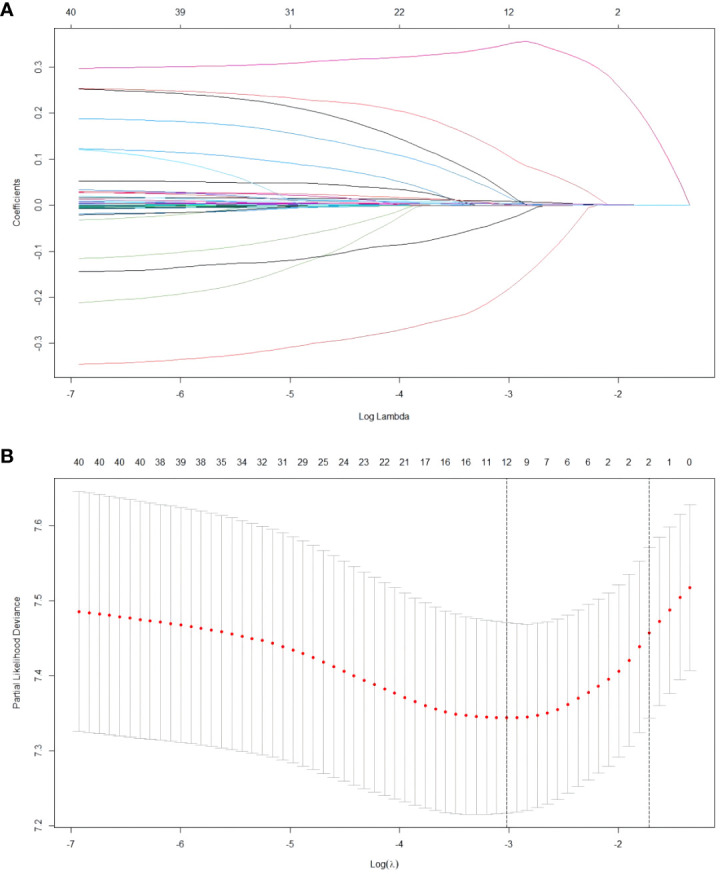
Results of the Lasso regression analysis in the training cohort. **(A)** The features pertaining to the variation of the variables coefficients. **(B)** The cross-validation utilized to determine the optimal value of the parameter λ.

The relationship between the patient’s clinical characteristics and RFS was evaluated by employing Lasso and Cox regression analyses. The Lasso regression was used in order to screen the parameters, and **Figure 1A** presents the variation features of the coefficients of these variables. The iterative analysis was subjected to the 10-fold cross-validation procedure, and when λ was 0.049 (Log λ= -1.309), a model with outstanding performance but the fewest possible variables was generated (**Figure 1B**). According to the results of Lasso regression analysis, age, gender, cirrhosis, BCLC stage, tumor number, tumor size, Lym, Alb, Glob, γ-GT, Palb and TT are factors significantly related to recurrence after ablation treatment in HCC patients. The factors significantly related to recurrence obtained by Lasso regression analysis were included in the multivariate Cox analysis, and it was indicated that there are still certain factors related to recurrence: age, gender, BCLC stage, tumor size, Glob and γ-GT (**Table 2**).

**Table 2:** Results of the multivariate Cox regression analysis in the training cohort.

	HR (95% CI)	P value
Age	1.015 (1.006–1.025)	**0.001**
Gender	0.766 (0.621–0.945)	**0.013**
Cirrhosis	1.169 (0.921–1.484)	0.198
BCLC stage	1.364 (1.111–1.675)	**0.003**
Tumor number	1.22 (0.958–1.553)	0.106
Tumor size	1.259 (1.021–1.553)	**0.032**
Lym	0.95 (0.844–1.068)	0.389
Alb	0.984 (0.963–1.006)	0.153
Glob	1.015 (1.001–1.031)	**0.048**
γ-GT	1.003 (1.001–1.004)	**<0.001**
Palb	0.999 (0.998–1.001)	0.47
TT	1.0019(0.965–1.038)	0.963

BCLC, Barcelona Clinic Liver Cancer; Lym, lymphocytes; Alb, albumin; Glob, Globulin; γ-GT, γ-glutamyl transpeptidase; Palb, prealbumin; PT, prothrombin time.

The bold values represent variables with a P-value less than 0.05.

### Nomogram built based on Lasso-Cox regression

**Figure 2:**
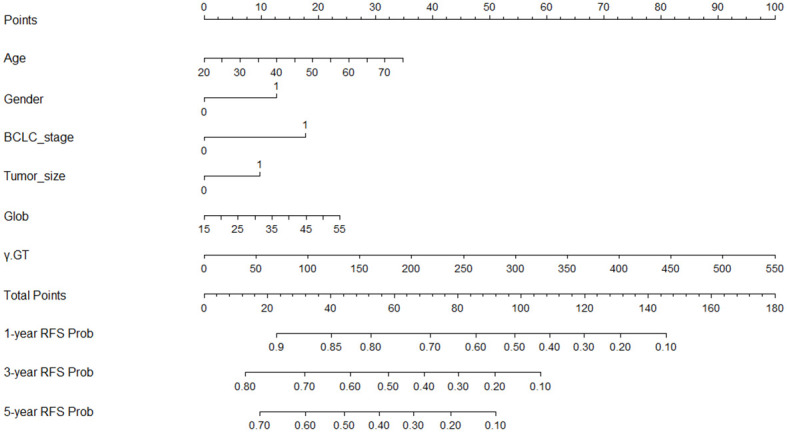
Nomogram built to predict 1-, 3-, and 5-year RFS in early-stage HCC patients treated with ablation. BCLC, Barcelona Clinic Liver Cancer; Glob, Globulin; γ-GT, γ- glutamyl transpeptidase; RFS, recurrence-free survival.

The above-mentioned six independent prognostic-related indicators screened out by Lasso and Cox analysis were integrated and a nomogram model based on these indicators was generated by running the data through the rms package of R software (**Figure 2**). Draw a vertical line corresponding to the value of each variable and intersect it with the Points axis in the first line, and the intersection point is the corresponding score value for that variable; Add the corresponding score values of each variable to obtain the total score; Draw a vertical line on the Total Points axis and intersect with the bottom probability axis to get the probability value corresponding to the total score. This value represents the probability of 1-year, 3-year and 5-year RFS for HCC patients after ablation treatment.

**Figure 3:**
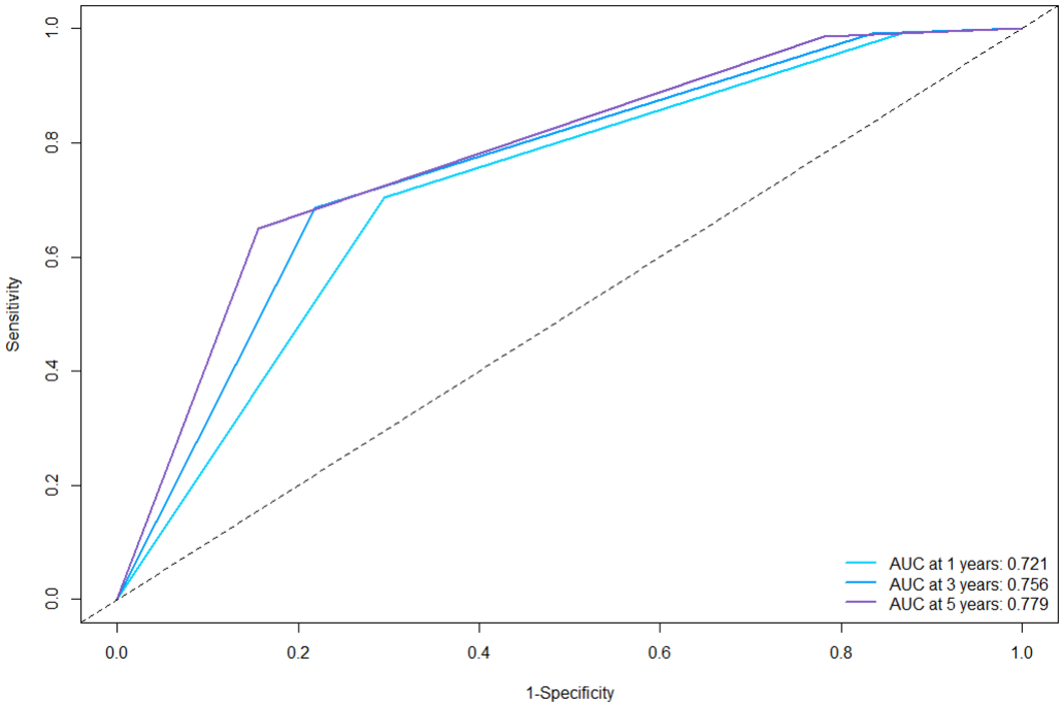
ROC curves of the nomogram for 1-, 3-, and 5-year RFS in the training cohort. ROC, receiver operating characteristics; AUC, area under the curve; RFS, recurrence-free survival.

In the training cohort, the C-index was 0.694 (95%CI: 0.670–0.717). Then we plotted the ROC curves of the 1-year, 3-year and 5-year RFS of the training cohort, and it showed that the AUCs of 1-, 3-, and 5-year RFS were 0.721, 0.756, and 0.779 (**Figure 3**). The AUC values of all three are greater than 0.7, indicating that the model has a good discrimination between tumor recurrence and non-recurrence individuals.

**Figure 4:**
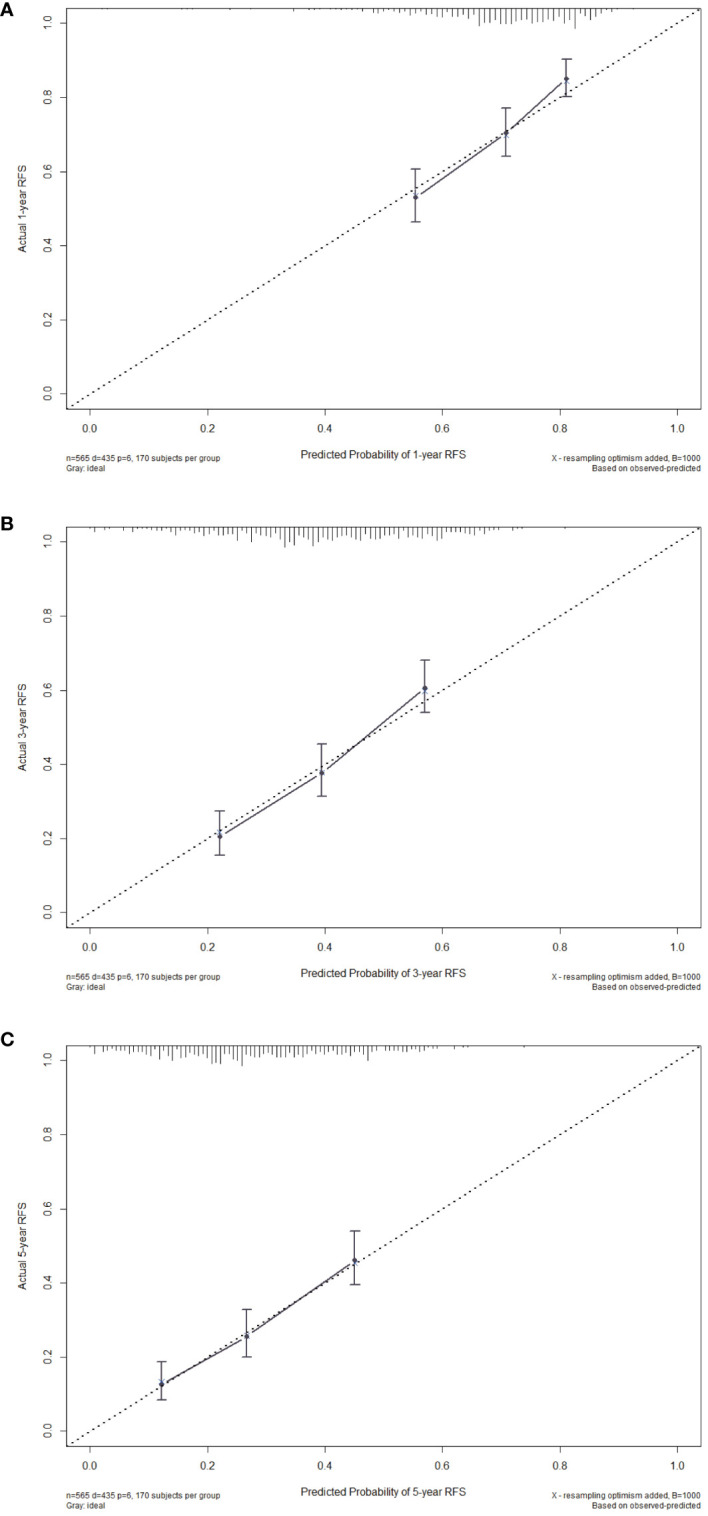
Calibration curves of the nomogram for 1-year **(A)**, 3-year **(B)** and 5-year **(C)** RFS in the training cohort. RFS, recurrence-free survival.

Then, we evaluated the consistency of the nomogram and drew the calibration curve of the training cohort (**Figure 4**). The X-axis represents the predicted RFS probability of the nomogram, the Y-axis represents the actual RFS, the dotted line on the diagonal represents the most ideal situation, and the solid line represents the calibration curve of this model. The closer the solid line is to the dotted line, the closer the model’s prediction results are to the ideal situation, indicating the higher the accuracy of the model. It can be seen from **Figure 4** that the calibration plot of the probability of 1-, 3-, and 5-year RFS showed that the nomogram prediction and actual observation were in good conformity with one another.

**Figure 5:**
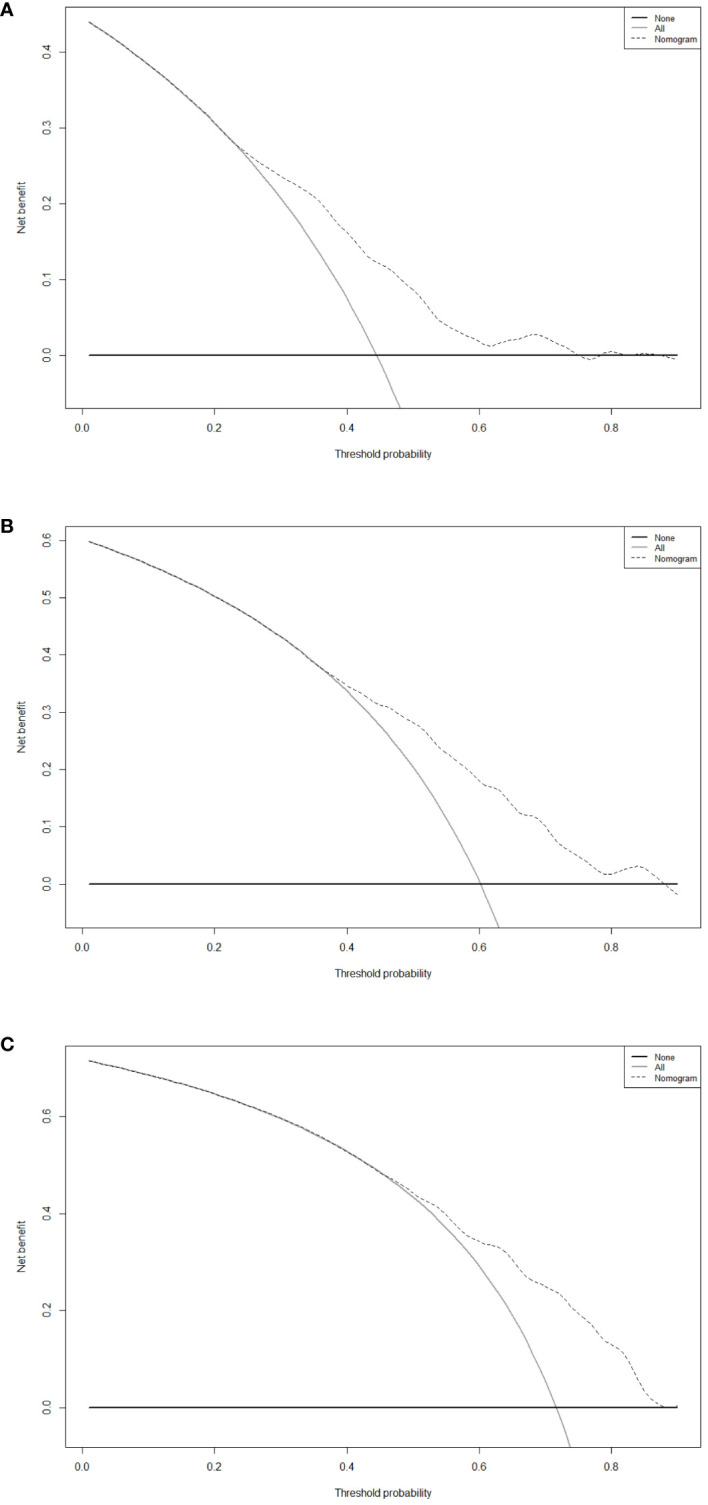
Decision curve analysis of the nomogram for 1-year **(A)**, 3-year **(B)** and 5-year **(C)** RFS in the training cohort. RFS, recurrence-free survival.

After evaluating the discrimination and consistency of the nomogram model, we drew DCA curves to judge the clinical effectiveness of the model and the net benefit rate of the model for patients, and the nomogram demonstrates significant net benefits within an appropriate threshold probability (**Figure 5**). The X-axis represents the threshold probability, and the Y-axis represents the patient’s net benefit.

**Figure 6:**
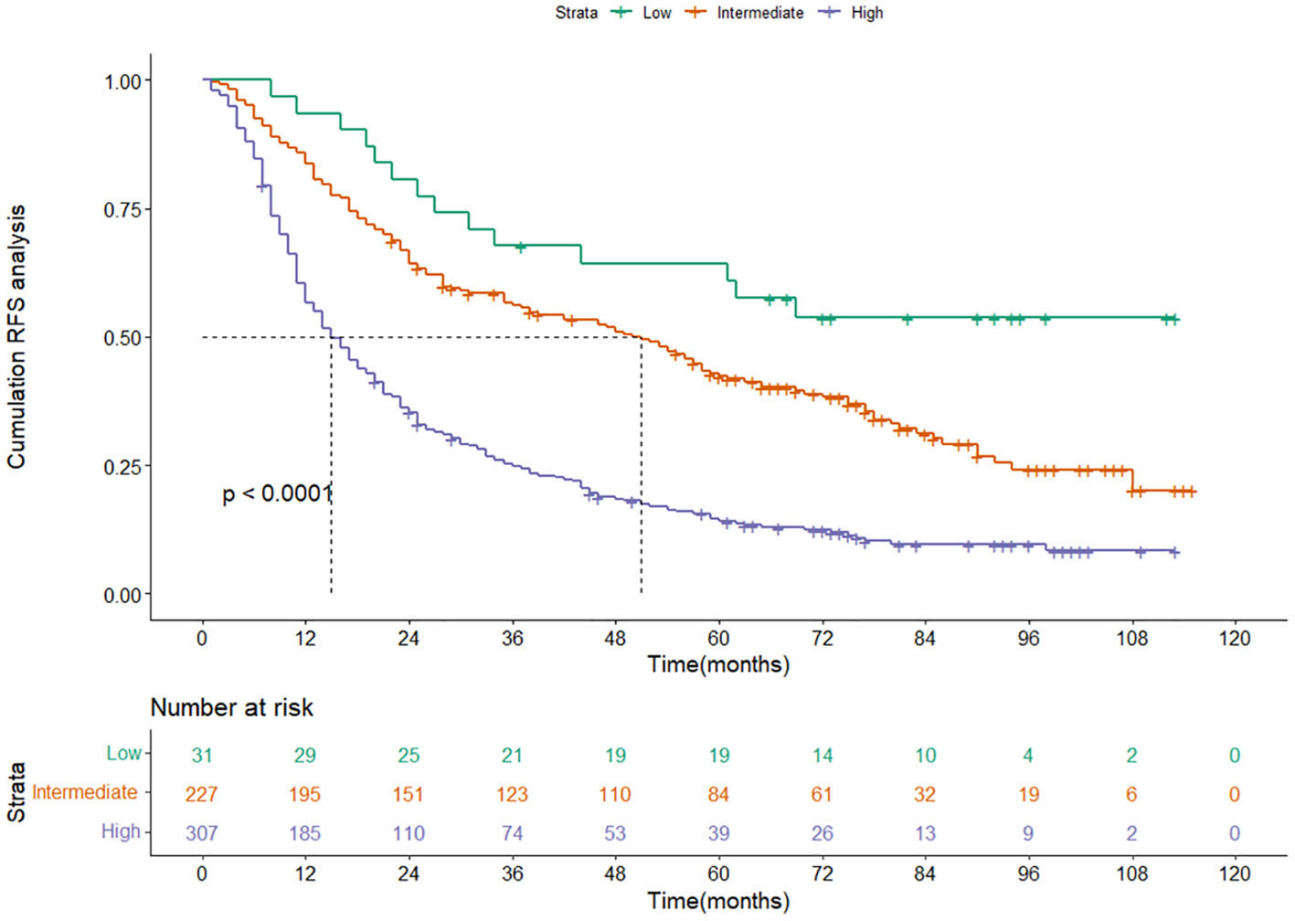
Kaplan-Meier curves of RFS for the low-, intermediate- and high-risk group in the training cohort categorized by the nomogram score. RFS, recurrence-free survival.

Based on the nomogram score, we divided patients into three risk groups: low-risk group, intermediate-risk group, and high-risk group. The RFS among different risk groups were statistically significant (P<0.001) (**Figure 6**), suggesting our model can effectively distinguish the recurrence risk in patients.

### Nomogram validation

In order to strengthen the reliability of the nomogram, we conducted an internal verification through the validation cohort. In the validation cohort, the C-index was 0.651 (95%CI: 0.617–0.684). The time-dependent ROC curves showed that the AUCs for 1- and 3-year RFS were 0.703 and 0.736, respectively, confirming that the nomogram can effectively distinguish between tumor recurrence and non-recurrence individuals (**Supplementary Figure S1**). The 1- and 3-year calibration curves proved the great accuracy of the nomogram (**Supplementary Figure S2**), and the DCA curves confirmed its outstanding clinical utility (**Supplementary Figure S3**). The Kaplan-Meier plots depicting the RFS for the low-risk, intermediate-risk, and high-risk groups in the validation cohort also demonstrated a significantly greater risk of recurrence in the high-risk group compared to both the low-risk and intermediate-risk groups (P< 0.001) (**Supplementary Figure S4**).

## Discussion

This study used the Lasso-Cox regression model to establish a nomogram for predicting recurrence in early-stage HCC patients treated with ablation. Compared with univariate regression, Lasso regression has stronger feature selection capabilities, better ability to handle collinearity, and the advantage of reducing model complexity. Compared with risk model formulas, the nomogram built based on the Lasso-Cox regression helps us visualize individualized morbidity risk assessment in the form of bar graphs, making it more intuitive and clearer to assess a patient’s recurrence risk, thereby enabling clinicians to design more effective follow-up and treatment strategies. For patients with a higher predicted risk of recurrence, intensified surveillance strategies, including more frequent imaging and biomarker evaluations, can be implemented to detect recurrences at an early, treatable stage. Conversely, patients with a lower risk of recurrence might benefit from less intensive follow-up, reducing the psychological and financial burden of over-surveillance. Furthermore, the nomogram’s insights can guide the intensity of adjuvant treatments post-ablation, ranging from more aggressive approaches for high-risk patients to conservative management for those with minimal risk. Patient counseling, too, can be significantly improved by providing individualized information about the risk of recurrence, thereby facilitating informed decision-making and setting realistic expectations for the disease course. This approach not only enhances the clinical management of HCC but also fosters a more patient-centered care paradigm, aligning treatment and follow-up strategies with each patient’s unique risk profile and preferences. The screened six factors are common examination indicators for HCC patients, and compared with other time-consuming and expensive examinations, the prediction model constructed with these common indicators can quickly score patients and provide certain reference value to guide personalized clinical decision-making for patients. In many previous studies, age, gender, BCLC stage, tumor size, Glob and γ-GT levels have been shown to be strongly associated with the survival and prognosis of HCC patients.

Age is one of the important factors affecting HCC and has a profound impact on its development and prognosis. Firstly, age-related physiological changes will affect the function of the immune system and reduce the body’s natural defense against tumors, making it more difficult to be attacked by the immune system, which reduces the impact of therapeutic modalities on tumors (23). Moreover, genetic and molecular mechanisms within tumors may change with aging, making tumors more complex and difficult to resist, and diminishing the effectiveness of treatment (24). For example, tumors from older patients demonstrate a significant increase in genomic instability, including somatic copy-number alterations (SCNAs) and mutations (25). Age-associated changes in DNA methylation, known as “epigenetic aging”, have been implicated in tumorigenesis (26). Secondly, as the patient ages, their physiological condition gradually declines. Their organ function begins to weaken, and the vitality of the immune system decreases, making it difficult for patients to bear the physical burden associated with surgical resection, ablation, TACE and so on (27, 28).

Gender is another independent risk factor for HCC recurrence. Our study shows that male HCC patients treated with ablation are more likely to experience recurrence. Previous studies have clearly demonstrated a significant difference in HCC recurrence rates between genders after treatment, and this finding has led to a discussion about how to better target male patients with more rigorous postoperative follow-up and monitoring strategies (29–32). This difference has long been thought to be related to estrogen levels, especially estradiol, which is considered a potential protective factor (33, 34). Estrogen is thought to affect the proliferation and differentiation of tumor cells through multiple pathways, and increases the rate of their apoptosis, thereby slowing down tumor progression (35). It is also considered to have anti-inflammatory and immunomodulatory effects, which can reduce inflammatory responses, inhibit the growth of tumor cells, and help the immune system fight tumor cells more effectively (36, 37). Moreover, androgen and androgen receptor (AR) also play significant role in promoting gender differences in the pathogenesis of HCC by affecting cell growth, differentiation and function (38). Additionally, the adipokine adiponectin, whose levels decrease in males and in obesity, plays a significant role in modulating HCC risk. The decrease in adiponectin correlates with increased liver cancer cell proliferation, indicating a link between metabolic health, adipose tissue function, and HCC risk (39). Therefore, female HCC patients may have a better prognosis after receiving ablation treatment.

BCLC stage is a widely adopted clinical staging system for HCC in the world. The main differences between BCLC stage 0 and A are the size and number of tumors as well as the state of liver function, and these differences directly affect the patient’s prognosis (40). Patients in BCLC stage A face a greater challenge because they may have more or larger tumors, which increases the difficulty of ablation. Moreover, some stage A patients may already have some impairment of liver function, which can reduce the effectiveness of treatment and may also lead to additional complications. The larger the tumor size, the higher the risk of HCC recurrence, which has also been confirmed in the past (27, 41–43). Large tumors are usually accompanied by a rich blood supply, which provides the tumor with sufficient nutrients and oxygen, further fueling its rapid growth. More seriously, this rich blood supply may provide a pathway for cancer cells to enter the circulatory system, thus increasing the risk of metastasis to other organs. It should also not be overlooked that large tumors may cause mechanical compression or injury to the surrounding normal tissues. This injury may lead to decreased liver function, which may affect the patient’s postoperative recovery and long-term prognosis.

The serum globulin level was recognized as a predictive risk factor for HCC patients after ablation treatment in this study. The most important function of globulin is participating in immune reactions as an antibody (44). Globulin levels may be affected when certain health problems occur in the body, so high globulin levels are often considered a precursor to certain diseases. For example, chronic inflammatory diseases are a common cause of elevated globulin levels, including chronic viral or bacterial infections, liver disease, kidney disease, and so on. Research has already shown that a sustained chronic inflammatory environment is an important contributing factor in a variety of tumors, with HCC being one of the cancers with which it is deeply associated (45). Studies in recent years have further revealed that long-term chronic inflammation is not only related to the development of HCC, but may also be associated with a poor prognosis for HCC patients (46, 47).

γ-GT may also be associated with poor prognosis in patients with HCC. γ-GT is an enzyme that catalyzes the conversion of γ-glutamyl and is mainly involved in the metabolism of glutathione, which maintains intracellular oxidative stress balance (48). In recent years, many studies have demonstrated the association of γ-GT with a variety of diseases, especially liver disease (49–51). Among them, the role of γ-GT in the prognostic assessment of patients with HCC has received particular attention. Carr et al. retrospectively analyzed a database of 470 HCC patients, which contained basic tumor parameters and survival-related data, and found that serumγ-GT was significantly associated with survival and tumor aggressiveness in HCC patients (52). Elevated levels of γ-GT may interfere with normal cell growth and apoptosis due to the fact that γ-GT is involved in a number of biochemical pathways associated with cell signaling, proliferation, and apoptosis.

Despite the established nomogram demonstrating promising predictive accuracy, our study is subject to several limitations. Firstly, as this study is a retrospective study, there may be some degree of selection bias. This bias arises because the study relies on existing records and data, potentially excluding relevant cases that were not documented or available for analysis. Consequently, the results may not fully represent the broader population or condition being studied. Moreover, the retrospective nature of the study could limit our ability to establish causality between observed outcomes and interventions or exposures, as we are looking back in time at what has already occurred. In the future research, prospective studies could be implemented to minimize selection bias. This approach involves enrolling participants prior to any intervention and tracking outcomes over time, thus providing stronger evidence for causality. Secondly, our study lacks external validation of the nomogram using independent cohorts from various centers. While the nomogram has been internally validated, validating it externally is critical for determining its generalizability and effectiveness across different clinical environments. Patient populations can significantly differ from one institution to another, and without external validation, the applicability of the nomogram in these varied settings remains uncertain. Future studies should aim to address this gap by conducting external validations, thus ensuring the nomogram’s relevance and utility in a wider range of clinical scenarios. Another limitation is the potential omission of additional risk factors that could significantly influence the risk of HCC recurrence. The complexity of HCC etiology suggests that other clinical, imaging, genetic, and environmental factors, beyond those currently analyzed, may significantly influence recurrence risk. Therefore, our findings and the predictive accuracy of the nomogram could be further refined by future research efforts dedicated to exploring these additional variables. This expansion of knowledge is essential for developing more comprehensive predictive tools that account for the multifaceted nature of HCC recurrence. Finally, our research was specifically designed to evaluate the outcomes of early-stage HCC patients undergoing ablation therapy. As such, the applicability of our findings to patients with advanced HCC remains to be determined and its predictive accuracy for patients receiving immunotherapy or anti-angiogenesis treatments, which are critical in the management of advanced HCC (53, 54), also remain untested. These limitations highlight the need for further research to explore the effectiveness and reliability of our nomogram across diverse patient populations and treatment modalities.

## Conclusion

This study developed a nomogram using Lasso-Cox regression to predict RFS in early-stage HCC patients following ablation, aiding clinicians in identifying high-risk groups for personalized follow-up treatments.

## Data availability statement

The original contributions presented in the study are included in the article/**Supplementary Material**. Further inquiries can be directed to the corresponding author.

## Ethics statement

The studies involving humans were approved by Ethics Committee of Beijing You’an Hospital, affiliated with Capital Medical University. The studies were conducted in accordance with the local legislation and institutional requirements. Written informed consent for participation was not required from the participants or the participants’ legal guardians/next of kin in accordance with the national legislation and institutional requirements.

## Author contributions

HZ: Conceptualization, Data curation, Writing – original draft, Writing – review & editing. SS: Formal analysis, Methodology, Writing – original draft, Writing – review & editing. WQ: Data curation, Formal analysis, Writing – review & editing. YS: Data curation, Writing – review & editing. RJ: Conceptualization, Project administration, Supervision, Writing – review & editing.

## Supplementary material

Supplementary Figure 1 ROC curves of the nomogram for 1- and 3-year RFS in the validation cohort. ROC, receiver operating characteristics; AUC, area under the curve; RFS, recurrence-free survival.

Supplementary Figure 2 Calibration curves of the nomogram for 1-year **(A)** and 3-year **(B)** RFS in the validation cohort. RFS, recurrence-free survival.

Supplementary Figure 3 Decision curve analysis of the nomogram for 1-year **(A)** and 3-year **(B)** RFS in the validation cohort. RFS, recurrence-free survival.

Supplementary Figure 4 Kaplan-Meier curves of RFS for the low-, intermediate- and high-risk group in the validation cohort categorized by the nomogram score. RFS, recurrence-free survival.
