# Supplementary figures and images for: Nomogram built based on machine learning to predict recurrence in early-stage hepatocellular carcinoma patients treated with ablation
Source: Front Oncol. 2024 May 10;14:1395329. doi: 10.3389/fonc.2024.1395329 (PMC11116608; doi:10.3389/fonc.2024.1395329)

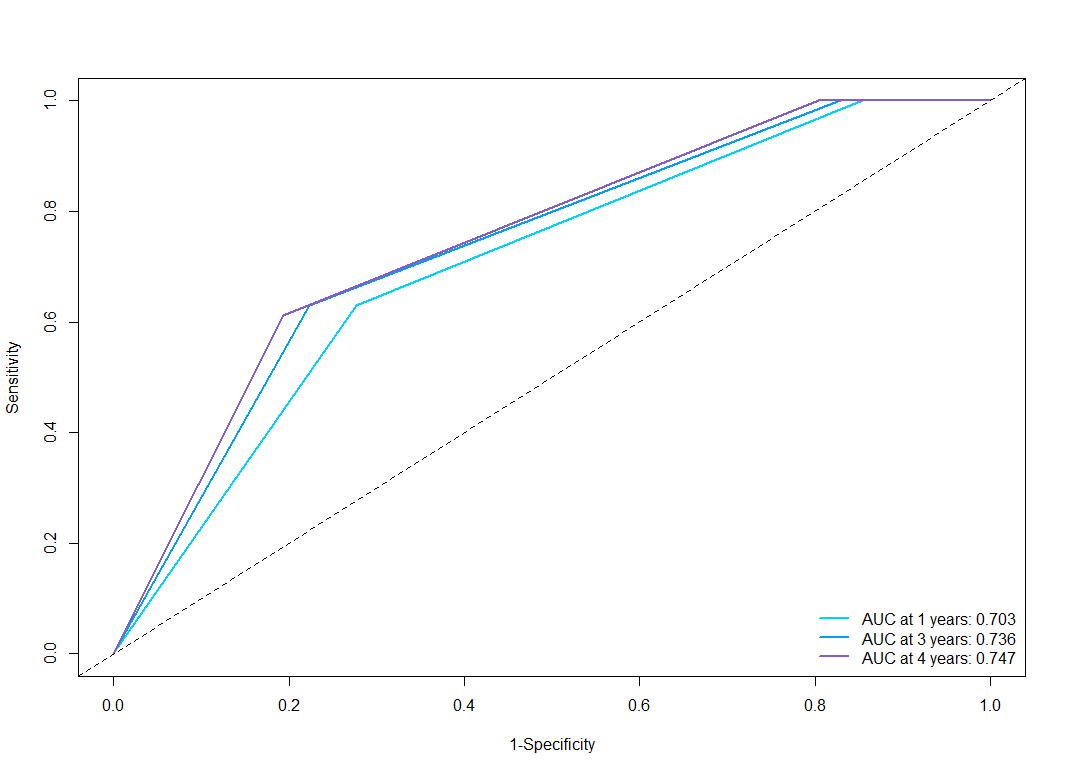

Supplement: Supplementary Figure 1 — ROC curves of the nomogram for 1- and 3-year RFS in the validation cohort. ROC, receiver operating characteristics; AUC, area under the curve; RFS, recurrence-free survival. [file Image_1.tiff]

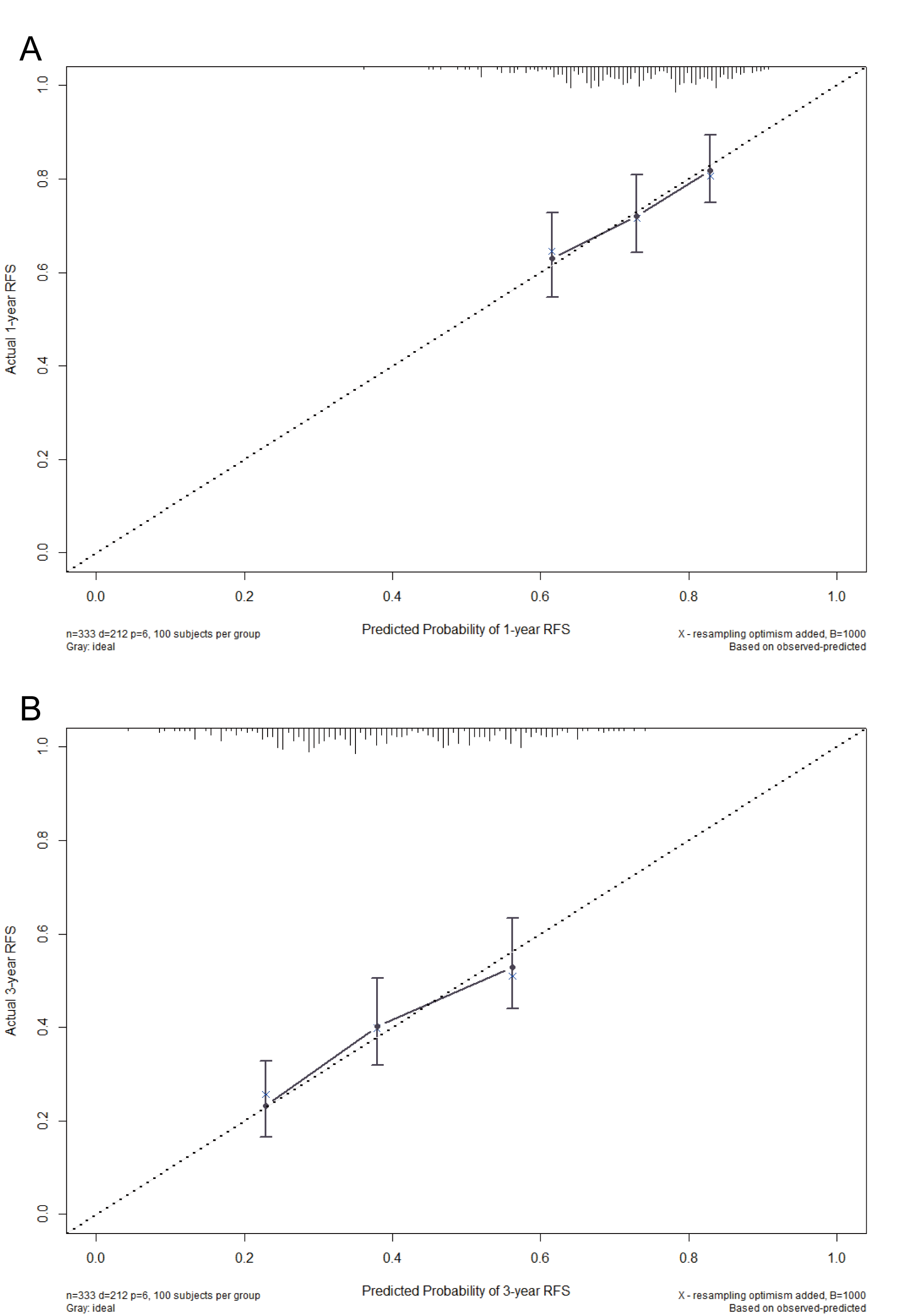

Supplement: Supplementary Figure 2 — Calibration curves of the nomogram for 1-year (A) and 3-year (B) RFS in the validation cohort. RFS, recurrence-free survival. [file Image_2.tif]

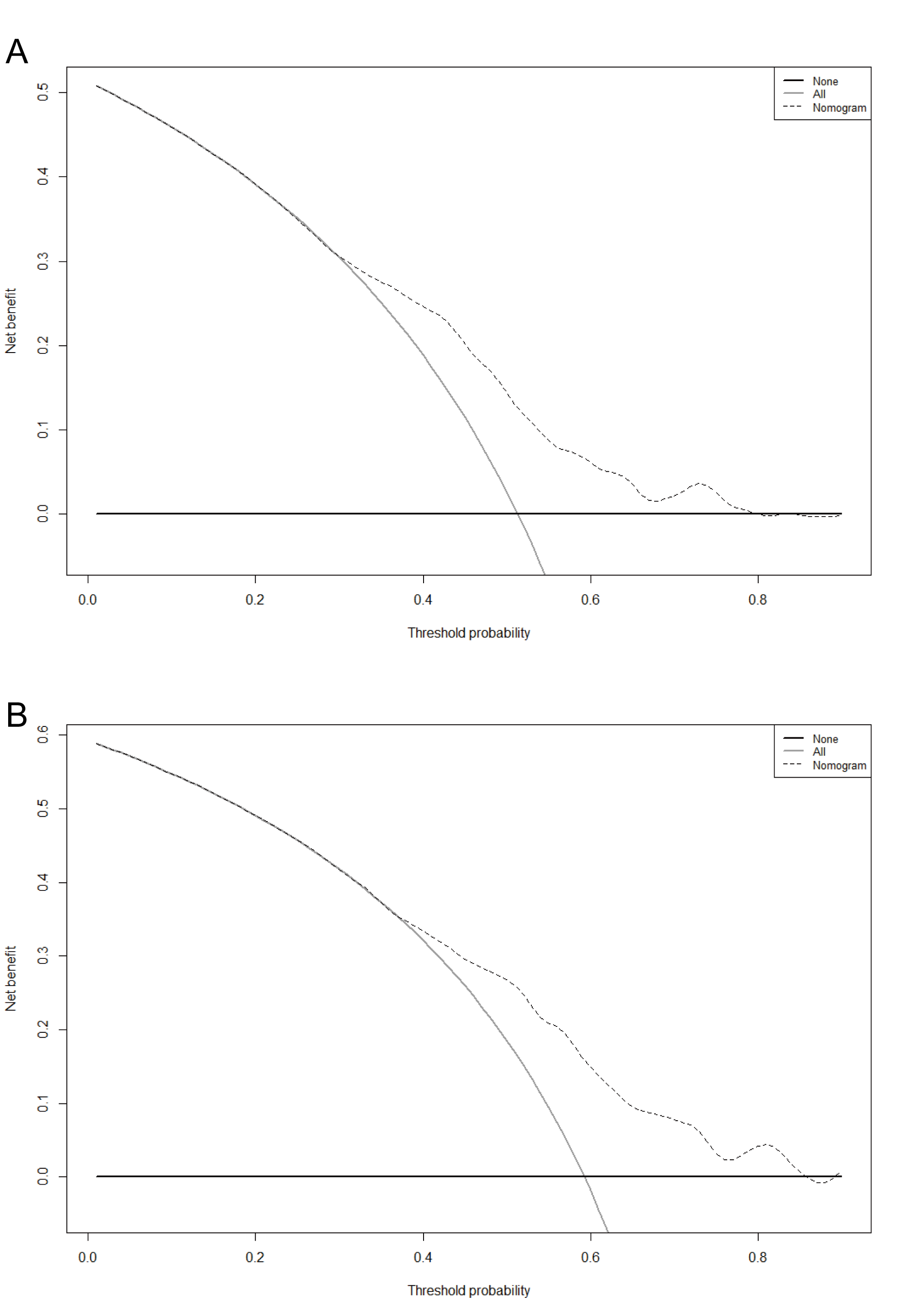

Supplement: Supplementary Figure 3 — Decision curve analysis of the nomogram for 1-year (A) and 3-year (B) RFS in the validation cohort. RFS, recurrence-free survival. [file Image_3.tif]

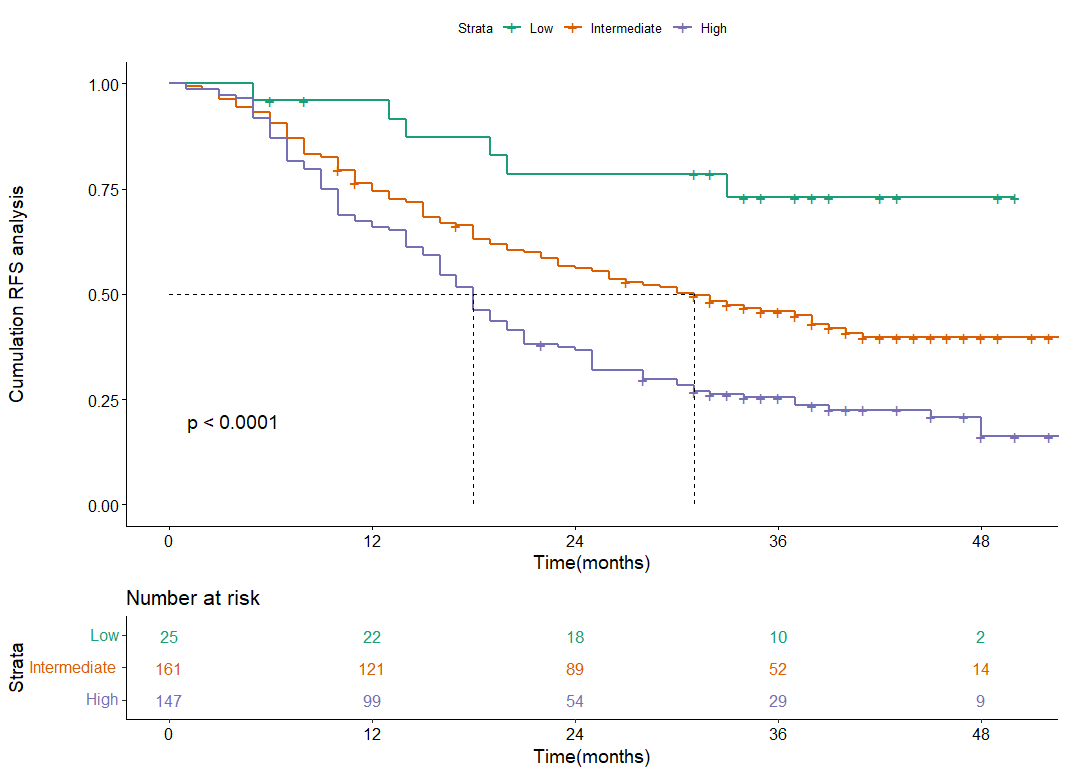

Supplement: Supplementary Figure 4 — Kaplan-Meier curves of RFS for the low-, intermediate- and high-risk group in the validation cohort categorized by the nomogram score. RFS, recurrence-free survival. [file Image_4.tiff]
